# Does the Wide Reach of the “Trauma-informed” Model Exceed its Narrow Grasp?

**DOI:** 10.1007/s11013-025-09938-z

**Published:** 2025-08-19

**Authors:** Vojtech Pisl, Sanne te Meerman, Allen Frances, Laura Batstra

**Affiliations:** 1 Department of Psychiatry, Faculty of Medicine in Pilsen, Charles University, Alej Svobody 76, 32300 Pilsen, Czech Republic; 2 Department of Child and Family Welfare, University of Groningen, Groningen, Netherlands; 3 Department of Psychiatry, Duke University, Durham, USA

**Keywords:** Trauma, Trauma-informed, Vulnerability, Iatrogenic harm, Reification, Trauma essentialism

## Abstract

Compared to the biomedical model of mental suffering, the increasingly influential trauma model has received little critical attention. We examine the discursive practices justifying and promoting the *trauma-informed care*: a set of assumptions and clinical recommendations presented as universal and uncontested guidelines for mental health practitioners. Critical review of two major guidelines for trauma-informed care – the *SAMHSA’s Concept of Trauma and Guidance for a Trauma-Informed Approach* (2014) and *A paradigm shift: relationships in trauma-informed mental health services* by Sweeney et al. (2018) – was inspired by critical discourse analysis and the analysis of reification. Trauma-informed care is a diverse set of recommendations combining generally accepted standards with the notion of broadly defined trauma as the primary cause of mental suffering. We have identified mechanisms that (1) present the broad trauma model as an assumption-free description of reality, (2) portray it as superior to other models, and (3) elevate the authority of those who adopt the broad trauma model to interpret the suffering of others. The discursive procedures found in the trauma-informed manuals are similar to those documented in the biomedical-psychiatric literature. Potential risks include iatrogenic harm, politicization of mental health care, and reduction of its diversity and effectiveness. Evaluation of the guidelines from the perspectives of safety, cultural validity, ethics, and cost-effectiveness should precede their implementation into clinical practice.

## Introduction

The trauma model of mental suffering serves as an inspiration for research and clinical practice alike. Its far-reaching implications range from the etiology (Mauritz et al., 2013; Stanton et al., 2020) and treatment (van der Kolk, 2014) of severe mental illness to sociopolitical movements (Brown, 2017; Figley et al., 2017). The trauma model is often embraced in psychiatry as an alternative to the dominant biomedical model, which has been called reductionist (Engel, 1977; Frances, 2013) and politicized (Beeker et al., 2023; Davies, 2017). Trauma has become increasingly popular in academic journals (Baes et al., 2023; Haslam & McGrath, 2020) and public resources (e.g., Maté & Maté, 2022; van der Kolk, 2014), even being dubbed “the word of the decade” by a popular magazine (Pandell, 2022). As its popularity grows, questions arise about potential reductionism and politicization of the trauma model.

The concept of psychological trauma is gradually expanding to include less severe and qualitatively new phenomena (Baes et al., 2023; Haslam & McGrath, 2020). These developments have given rise to at least two qualitatively different conceptions of trauma: a scientific and clinical category, which we will refer to as *narrow trauma,* and a sociopolitical and moral category, which we will refer to as *broad trauma.* Narrow trauma indicates a specific set of extraordinary events defined by a *gatekeeping criterion*^1^ that are above the capacities of the subject and are followed by related negative consequences such as persistent avoidance of stimuli and associated intrusions (American Psychiatric Association, 2013; Krupnik, 2019). Broad trauma, on the other hand, encompasses all negative consequences of any event or situation (Krupnik, 2019). Some scholars prefer the latter to acknowledge the suffering of those who are not traumatized in the narrow sense (e.g., Alexander, 2004; Avina & O’Donohue, 2002). Others criticize this perspective, arguing that it renders the concept of trauma meaningless, because it could classify any adversity as trauma (e.g., Krupnik, 2019; Rosen, 2004).

The severity of narrow trauma and the prevalence of broad trauma combine in a powerful cultural idiom. As neatly summarized more than a decade ago, “the concept of a person in contemporary Western culture now emphasizes not resilience but vulnerability” (Summerfield, 2008, p. 234). This development is welcomed and promoted, especially by proponents of the *trauma-informed care*, who call for the establishment of the trauma model as the universal model of mental suffering and the presumption of trauma in every individual (Substance Abuse & Mental Health Services Administration, 2014; Sweeney & Taggart, 2018; Sweeney et al., 2016, 2018). Others express that promoting the broad trauma model can cause harm by encouraging trauma interpretations at the expense of more positive alternatives, through self-fulfilling prophecies and by removing agency from service users (Lester, 2013; Summerfield, 2022).

Accepting trauma as an explanation of mental suffering is an arbitrary choice. Mental suffering is inherently complex and multicausal, as explained by the biopsychosocial model (Engel, 1977) or nonlinear conceptualizations of psychopathology (Olthof et al., 2023; Schmittmann et al., 2013). Only the limits of human cognition led us to reduce its complexity by using models, such as the biomedical model or the trauma model. The literature on the biomedical model has been criticized for reifying socially constructed disorders: presenting them as if they were tangible, physical diseases, and failing to acknowledge the shortcomings of such explanations (e.g., Batstra et al., 2017; Beeker et al., 2023; Hyman, 2010; Korteling, 2014; Schleim, 2022; te Meerman, 2019; te Meerman et al., 2022). Such reification of human suffering, defined as “converting a set of various human experiences (…) into the notion of a single identifiable derailment existing independently of the observer, separable from the subject and having its own agency, causes and consequences,” is almost exclusively studied in relation to biomedical explanations (Pisl et al., 2025). The current study therefore aims to examine whether similar discursive mechanisms that reify psychiatric constructs and evade the limitations of the medical model (e.g., suggestive or imprecise language choices, logical fallacies, or textual silence about contradicting evidence; Pisl et al., 2025; te Meerman, 2019; te Meerman et al., 2017a, 2017b; te Meerman et al., 2022) are also at play in resources that build on the trauma paradigm: the trauma-informed guidelines.

## Methods

### Data Selection

To increase ecological validity and mimic a practitioner’s search for information, we selected manuals that were most readily available using Google. We searched for two documents that were (1) recent (less than a decade old), (2) influential (clearly valued by the professional community as indicated by large number of citations compared to other similar documents), (3) published by an authority, (4) provided universal clinical recommendations (i.e., recommendations that were not limited to the treatment of individuals traumatized in the narrow sense), and (5) comprehensive. Specifically, in November 2023, we selected the top result returned by Google Scholar for the keywords “trauma-informed, mental health” and the first result returned by Google Search for the keywords “trauma-informed mental health pdf” (after an initial search for “trauma-informed mental health” resulted in a web presentation that was judged to be too brief and lacking authority). Both results, hereafter referred to as SHIFT and SAMHSA, were current and influential, written by clinical experts, published by reputable resources, and providing comprehensive recommendations:

By “SHIFT,” we refer to *A paradigm shift: relationships in trauma-informed mental health services* (Sweeney et al., 2018) identified by the Google Scholar search. The guidelines are (1) recent (published within the last decade), (2) influential (returned as the first result for the search phrase, cited by more than one hundred peer-reviewed studies according to the Google Scholar), (3) published by an authority (a peer-reviewed journal by the Cambridge University Press), (4) making universal clinical recommendations (i.e., recommendations that are not limited to the treatment of those traumatized in the narrow sense; presented as universal, authoritative recommendations – e.g., by including “learning objectives” and multiple-choice test), and (5) comprehensive (providing an overview of the topic rather than focusing on a specific detail – e.g., “Gain an understanding of what trauma-informed approaches are and why they have emerged, including the potential for (re)traumatisation in the mental health system,” p. 319).

By “SAMHSA,” we refer to *SAMHSA’s Concept of Trauma and Guidance for a Trauma-Informed Approach* (Substance Abuse & Mental Health Services Administration, 2014), as identified by the Google Search. The guidelines are (1) recent (published within the last decade), (2) influential (returned as the first result for the search phrase, cited by more than one hundred peer-reviewed studies according to the Google Scholar), (3) published by an authority (authored by the US federal *Substance Abuse and Mental Health Services Administration*), (4) making universal clinical recommendations (i.e., the manual is intended to inspire “child welfare, education, criminal and juvenile justice, primary health care, the military and other settings that have the potential to ease or exacerbate an individual’s capacity to cope with traumatic experiences,” aiming to “guide systems to become trauma-informed” – p. 3), and comprehensive (providing an overview of the topic rather than focusing on a specific detail – e.g., “The purpose of this paper is to develop a working concept of trauma and a trauma-informed approach and to develop a shared understanding of these concepts that would be acceptable and appropriate across an array of service systems and stakeholder groups,” p. 3).

## Analysis

Our critical review of the manuals was inspired by the principles of critical discourse analysis (CDA) and the analysis of reification. Specifically, our analysis was inspired by the applications of elements of CDA in psychiatry to study how models of mental suffering and disorders are reified (te Meerman et al., 2017a, 2017b; te Meerman, 2019; te Meerman et al., 2022) and established as dominant (Adam et al., 2023; Freedman & Honkasilta, 2017). A recent conceptual analysis showed that while these discursive strategies are typically studied in relation to the biomedical model, the same strategies can be used in other models, such as those that interpret human suffering as trauma (Pisl et al., 2025). Thus, our analysis focuses on the use of language that may present one interpretation of human suffering as superior to its alternatives, while portraying itself as a neutral description (Wodak & Meyer, 2009).

The analysis involved iterative processes of coding and grouping the linguistic elements of interest. During repeated readings of the manuals, we focused on identifying linguistic elements characteristic of reification and/or discursive strategies establishing dominance. First, we focused on identifying possible reification processes that can transform an abstract, descriptive model into a notion of a real entity with its own independent existence and agency, often using circular logic (Pisl et al., 2025; te Meerman et al., 2022). Second, we have focused on whether and how such reification or other discursive processes may assert the dominance of the model underlying the trauma-informed care. Repetitive coding and grouping of the elements continued until the authors agreed that the structure of the results provided a meaningful overview of the discursive practices of interest.

## Results

### Overview

Both manuals build on the broad trauma model, which is presented as neutral and universal. Trauma is defined as “an event, series of events, or set of circumstances that is experienced by an individual as physically or emotionally harmful or life threatening and that has lasting adverse effects on the individual’s functioning and mental, physical, social, emotional, or spiritual well-being” (SAMHSA), with poverty, colonialism, or urbanization cited as examples of harmful events (SHIFT). The manuals do not include the gatekeeping criterion (i.e., traumatization is not limited with respect to any specific quality of the exposure other than being consequential and perceived as harmful). Based on this definition, trauma is presented as widespread, and recommendations are made to approach each individual as a potential trauma victim to avoid retraumatization. The manuals introduce a set of principles for clinical practice to ensure that all service users and providers receive the care a traumatized individual may need. They include a wide range of recommendations from procedures routinely used in mental healthcare to adopting the assumption that human lives are primarily determined by sociopolitical phenomena.

We identified several discursive practices that present the broad trauma model as ideologically neutral and professionally uncontroversial and promote it among healthcare users and providers. The practices, summarized in Table 1, are categorized into three areas according to their function and are discussed below in light of further literature. The first area includes practices that present the broad trauma model underlying trauma-informed care as a neutral description of reality, examples of which are included in Table 2. The second consists of practices that present the broad trauma model as superior to other models (see Table 3 for examples). The third consists of recommendations to help disseminate the model to mental health service providers and users (Table 4).

**Table 1:** Key discursive practices relevant for justifying and promoting the trauma-informed care

Portraying reality as trauma-centered	Terminological inconsistency	Treating the broad trauma, narrow trauma, and adverse experience (traumatizing or not) as if they were one construct
	Reification	Treating the model underlying trauma-informed care as if it was an objective description of reality, resulting in circular reasoning
	Textual silence	Omitting data, arguments, constructs, theories, and critiques that problematize the model underlying trauma-informed care
Portraying broad trauma as superior to other models	False dichotomies	Presenting a forced choice between two options when in fact the options are not contradictory and/or more than two options are available
	Persuasive fictionalization	Contrasting fictional narratives about idealized trauma-informed treatment with stories about failures in standard mental healthcare
Promoting the notion of broad trauma	Argumentation from authority	Presenting those who have adopted the broad trauma model as if they have a special authority to interpret the mental suffering of others
	Persuading care users and providers to adopt the model	Requiring all mental healthcare providers to adopt a set of beliefs and perspectives. Recommending measures that motivate service users to view themselves as traumatized

**Table 2:** Examples of inconsistent meaning of the term “trauma”

Different constructs denoted by the term"trauma"	Selected Excerpts from the SHIFT
Formal definition:"trauma"refers to the broad trauma	*"Trauma is an external event with long-lasting effects on well-being. It can include real, or perceived threat (…) Less well-understood forms of trauma include racism, urbanicity, poverty, inequality, oppression and historical trauma (the legacy of entire groups having experienced violence such as slavery, the Holocaust or genocide)"*
The formidability of trauma:"trauma"refers to the narrow trauma and/or objective adverse childhood experiences and abuse	*“Neuroscientific research has demonstrated the impact of trauma on the brain, including changes to the sensory systems, gray-matter volume, neural architecture and neural circuits (e.g., Read *2014*).”* The study by Read (2014) is a review of literature discussing the traumagenic neurodevelopmental model, using human and animal data related to traumatization in the narrow sense or objective childhood adverse events
	*"[C]hildhood trauma... can shorten life expectancy (e.g., Felitti 1998)."* The study by Felitti (1998) quantifies the effects of children abuse
The prevalence of trauma:"trauma"refers to adverse experiences	*"Research has consistently found that people using mental health services have experienced high rates of trauma in childhood or adulthood (e.g., Kessler 2010)."* The study by Kessler (2010) quantifies the prevalence of objective adverse childhood experiences (incl., for instance, divorce or financial hardships) that may or may not have been experienced as traumatizing

## Portraying Reality as Trauma-Centered

A common discourse practice in establishing a discursive dominance is to present a perspectival stance as an objective, non-ideological description of reality (Freedman & Honkasilta, 2017). Two aspects were identified that create such a trauma-centered reality: inconsistent terminology and reification of the trauma model. Regarding the latter, the use of the word “trauma” interchangeably for broad trauma, narrow trauma, and adverse experiences creates a conflated view on the prevalence and severity of traumatization. Trauma is defined broadly, implying a wide range of trauma. Studies that estimate the prevalence of common adverse experiences are used to document the large prevalence of trauma. At the same time, studies measuring the effects of narrowly defined severe traumatization are cited to demonstrate the severity of the effects of trauma (see Table 2 for specific examples). As a result, the severity of narrowly defined trauma is generalized to those portrayed as victims of the ubiquitous broad trauma.

Confusing the trauma model with reality itself leads to circular reasoning, a hallmark of reification (Glas, 2020; te Meerman et al., 2022). For instance, the manuals cite the high prevalence of trauma as justification for adopting the trauma-informed perspective. Indeed, when reality is interpreted through a model that “centralizes the causal role of trauma” (SHIFT), trauma becomes ubiquitous. Yet, its ubiquity reflects the observer’s decision to interpret any adverse outcome as a trauma rather than the prevalence of specific events. Consequently, justifying the need for the trauma perspective by the finding that trauma is ubiquitous from the broad trauma perspective is a circular argument.

Similarly, the same circularity problematizes the argument that the trauma perspective is needed because trauma is “harmful and costly public health problem” (SAMHSA). With trauma defined as any event or situation that has adverse consequences, anything that is costly and harmful is trauma. Thus, the claim that trauma is harmful and costly is tautological. This tautology then shifts the perceived cause of the harm and costs from the event or situation causing them to the traumatization itself. Indeed, statements such as “trauma can affect families, groups…” (SAMHSA) or “trauma can influence emotions and therefore behavior” (SHIFT) reflect the observer’s choice to see trauma as the ultimate cause of human hardships. The relationship between adverse circumstances and their consequences can be explained and addressed directly: for example, “poverty can affect families, groups” or “urbanicity can influence emotions and therefore behavior.”

Finally, data and arguments unfavorable to the broad trauma model are absent from the manuals, resembling the discursive practice of textual silence, common in reification (Machin & Mayr, 2012; te Meerman et al., 2022). For example, the manuals present traumatization as the default response to an exposure, despite the heterogeneity of human responses to adversity (Boals, 2018; Bonanno, 2008). Similarly, they do not include perspectives from research on post-traumatic growth or cultural factors that shape whether or not an individual experiences a situation as traumatizing. Finally, while clinical and administrative procedures are recommended, their key aspects such as quality of evidence, safety, efficacy, cost-effectiveness, or compliance with ethical and legal requirements and generalizability are not discussed.

## Portraying Broad Trauma as Superior to Other Models

The manuals present the trauma model as superior to other models by using presuppositions, false dichotomies, and by replacing argumentation with value-laden stories. While advocating diversity, the manuals promote the broad trauma model at the expense of other perspectives. This is best reflected in the call for “a holistic model of mental distress that centralizes the causal role of trauma” (SHIFT), making no distinction between holistic and trauma-focused interpretations of reality.

Dichotomies are often presupposed, such as between the trauma model and the “current mental health system” (SHIFT). The “current system” is presented as uncaring and harmful because it allegedly fails to acknowledge that psychopathology can develop as a response to a stressor, and trauma-informed care is offered as the only alternative. However, current views of psychopathology acknowledge the adaptive functions of psychopathology (e.g., Andrews & Thomson, 2009; Swanepoel et al., 2017). Hence, the interpretation of human behaviors as adaptations to present and past situations is more in line with the “current system” than in opposition to it. Similarly, the trauma model is not needed to acknowledge the relationships between mental health and what is included under the umbrella term *trauma* in the manuals, such as urbanicity or poverty.

A powerful dichotomy between “trauma-informed” and “trauma-uninformed” is created by SHIFT presenting the trauma-informed care as the best or only viable option. Constructs as “trauma-uninformed” care, staff, and organization are introduced as opposites to trauma-informed care (SHIFT). No definition or evidence is provided for the newly introduced construct. According to its abstract, the SHIFT describes “how service users can be retraumatized by ‘trauma-uninformed’ staff.” However, no evidence or explanation is provided that would link retraumatization to “trauma-uninformed” (but not “trauma-informed”) staff^3^. For example, the risk of retraumatization is described primarily through a story in which a suicidal patient named Claire is overwhelmed by being constantly monitored by staff who “do not interact with her much” and provide her with “few opportunities to talk about the things that led her to feel suicidal.” Then another story is presented in which the patient feels hopeful because “staff are as interactive as possible. They attempt to validate Claire’s feelings of distress.” These stories provide a compelling narrative example of how failure to communicate with a patient can lead to adverse outcomes. However, implementation of trauma-informed guidelines is neither a necessary nor a sufficient condition for patients to feel hopeful rather than frightened.

The trauma-informed guidelines consist of a heterogeneous set of recommendations, leaving considerable room between full adherence to the guidelines and being “trauma-uninformed.” Some of the recommendations are generally accepted and not specific to the broad trauma model (e.g., valuating patient individuality, ensuring care provider transparency, fostering collaboration and trust, prioritizing patient safety). Other recommendations relate to the clinician’s worldview (e.g., adopting the “broad-based understanding of trauma … involving an appreciation of community, social, cultural, and historical traumas,” SHIFT). “Trauma-uninformed” care is then portrayed as suboptimal or even iatrogenic in fictional and/or carefully selected^4^ vignettes describing failures to adhere to generally accepted good practices (SHIFT). These failures are then contrasted with the idealized trauma-informed care: The vignettes present the trauma-informed care as appropriate and compassionate, while examples of inappropriate and uncompassionate care are presented as the “current system.” For instance, in one of the vignettes, the patient Emma tells her psychiatrist that “she would like to be in touch with women who are going through similar experiences.” The psychiatrist, however, overhears her request and prescribes her medication instead, leaving her “feeling confused and guilty.” In a complementary vignette, a trauma-informed professional “links with Emma's health visitor to see what local or online peer support options there are. Emma realizes that what she is feeling is common in her circumstances and feels connected enough with others.”

Both manuals emphasize the collaborative role of trauma survivors as co-authors. A dichotomy is presupposed between trauma survivors and others. Moreover, appeals to “survivor’s perspective” (SAMHSA) as a single point of view assume homogeneity of those who have experienced traumatizing events. Yet, the majority of those exposed to events classified as potentially traumatic do not perceive such events as trauma (Boals, 2018). The perspective of “trauma survivors” is presented as superior to the knowledge of individuals who may have experienced the same event but who self-identify as “survivors of depression” or “survivors of human misery” or who do not consider themselves as survivors at all, despite having faced comparable circumstances. According to the definitions presented in the manuals, any event that is perceived as harmful and causally linked to lasting negative consequences is considered a trauma. Thus, the survivor status is based on a decision to adopt a particular interpretation of one’s hardships^5^. Excluding the personal testimonies and professional positions of others than those with a trauma survivor perspective allows the call for guidelines based on one interpretation of reality to be “implemented for all service users, regardless of whether they have survived trauma” (SHIFT).

## Spreading the Broad Trauma Narrative

Finally, implementing the measures proposed by the manuals would, in effect, mean actively disseminating the broad trauma model. The justification of mental healthcare guidelines with terms such as “survivor knowledge” (SAMHSA) assumes that the authority survivors have over their individual idiosyncratic experience is transferable to authority over clinical guidelines. By promoting “trauma survivors” as peer counselors and sources of knowledge, the manuals imply that those who choose the trauma model as the explanatory framework for their hardships should have a privileged position in the discourse. For instance, the SHIFT states that “This article, authored by trauma survivors and service providers, describes trauma-informed approaches to mental healthcare… [T]rauma does not need valid and reliable diagnosis or measurement, because principles of engagement are implemented for all service users, regardless of whether they have survived trauma.” Similarly, the SAMHSA states that “Peer support and mutual self-help are key vehicles for establishing safety and hope, building trust, enhancing collaboration, and utilizing their stories and lived experience to promote recovery and healing. The term “Peers” refers to individuals with lived experiences of trauma.” The statements assume that the status of a trauma survivor grants unique authority in developing and providing mental healthcare services, including for populations who do not conceptualize their distress as trauma.

Furthermore, the manuals require care providers to interpret the reality through certain ideological constructs, such as the gendered and racialized society or predetermination of psychopathology by economic and historical context, by including “racism, urbanicity, poverty, inequality, oppression, and historical trauma” as exemplar cases of trauma. Some individuals throughout the Western as well as other cultures may find these requirements incompatible with their cultural beliefs: for instance, groups of certain political orientations (e.g., those who prefer to believe they live in a liberal society and adversities are not primarily externally induced), lifestyle choices (e.g., those who prefer to see urbanicity primarily as an asset rather than a burden to human well-being), philosophical worldviews (e.g., those who prefer to interpret their lives as primarily determined by their actions over historical events) or esthetic/ethical perspectives (e.g., those who find it uncongenial to extend the medical term traditionally reserved for the exposure to extreme, lethal events).

The manuals suggest that service users should be motivated by clinicians to adopt these beliefs and perspectives. A prophylactic trauma assessment is recommended for each person seeking support. The recommendation to ask every service user about the broadly understood traumatization (such as history of poverty, urbanization, or potential cultural trauma) would, per se, imply to the service users that their problems should be viewed primarily through the lens of broad trauma. For instance, the SHIFT recommends to “ask everyone about their experiences of trauma and abuse,” “preface trauma questions with a brief normalizing statement,” and “reassure the person that disclosure is a good thing.” These procedures may further motivate service users to disclose trauma as an act of compliance, whether or not they feel traumatized. Thus, these recommendations increase the sensitivity of trauma assessment at the expense of its specificity.

Finally, implementation of these recommendations may bias clinicians’ experience through self-fulfilling prophecies. If clinicians encourage service users to interpret their hardships as trauma, service users will be more likely to do so. As a result, clinicians may encounter more seemingly traumatized patients than they would if they had not encouraged the trauma interpretation. This effect may be particularly strong because most people in Western societies who have experienced an event classified as potentially traumatic have not perceived it as such (Boals, 2018; Bonanno, 2008).

## Discussion

The trauma-informed care manuals most available to a practitioner present the broad trauma model underlying trauma-informed care as neutral, assumption-free, uncontroversial, and superior to other models. Several discursive practices help achieve this, including assuming the position of epistemic authority, presenting the model as reality, and textual silence about its limitations, data, and arguments that challenge the model. Although problematic, such practices are common in presentations of various models of psychopathology (Pisl et al., 2025) and have been documented particularly in the biomedically oriented literature (e.g., Batstra et al., 2017, 2021; Freedman & Honkasilta, 2017; Hyman, 2010; Kinderman et al., 2013; Patten, 2015; Stoyanov, 2020; te Meerman, 2019; te Meerman et al., 2017a, 2017b, 2022). Additional risks of these practices, which are largely specific to the broad trauma model, are discussed in the following sections.

## The Risk of Misconceptions About Trauma due to Terminological Inconsistency

Terminological ambiguity and reification can lead to invalid assumptions about trauma. The manuals use the term *trauma* to refer to different concepts, ranging from the experience of adversity to broad traumatization to narrow trauma. This can lead to invalid generalizations of the clinical status of those with narrow trauma or Post Traumatic Stress Disorder (PTSD) to anyone who has experienced an adverse event. Similarly, the use of the term “trauma” as a name for different constructs may lead to confusion. Without additional specification, it is not clear when the term refers to clinical assessment of functioning, humane recognition of human suffering, or sociopolitical, legal, or moral decisions about responsibility and blame. Importantly, moral responsibility is not fully congruent with causal responsibility (Talbert, 2023). Failure to appreciate the difference between these two constructs can problematize healing when a victim is mistakenly assumed to be traumatized or powerless based on his or her moral or legal victimhood. Moreover, if traumatization in the clinical sense is linked to injustice, medical care may depend upon the care provider’s judgment about who should be held responsible for past events rather than on the needs of the care recipient.

Furthermore, presenting trauma as an objective entity independent of culture may misguide societal measures. For instance, Read and colleagues (2014) claim that “if the six types of child adversity studied were to disappear, a third of new psychosis cases would be avoided.” By these six types of adversity, they refer to constructs measured in a previous study, including experiences as “parental harshness,” “failing to provide food or clothes,” or “exposure to behavior that might result in trauma” (Varese et al., 2012). Relative phenomena such as poverty or parental harshness, however, cannot just disappear, because intervening in one case changes the norm for the others. Buying expensive clothes for one child would reduce the relative value of the clothes of all the others; making the parents of one child less harsh would increase the relative harshness of the parents of all others. Such relativity, however, is not always recognized in the trauma literature, resulting in misleading notions of an adversity-free society.

## The Risk of Tribalism in Clinical Care due to Politicization of Trauma

The ideologization of health care risks turning professional debates into tribal conflicts. When clinical guidelines impose ideological positions from a position of authority, clinicians and researchers may divide into opposing camps, engaging in mutual attacks rather than collaborating to foster a fair and stimulating competition of ideas and evidence. Such attacks can already be observed, for example, in debates about the politicization of psychoanalysis (Burston & Nelson, 2023; Kemp, 2023). Extensive ideologization of mental health education has been documented in the United Kingdom (Sherwood & Miller, 2022), leading to calls to protect patients from the harms of politicized mental healthcare (Save Mental Health, 2022). Such politicization of mental healthcare can discourage help-seeking by individuals who do not agree with the clinicians’ ideology and undermine public trust in healthcare institutions.

Two aspects of the manuals may increase the risk of similar skirmishes in the field of trauma. First, the manuals impose ideological requirements that may increase the risk of politicizing clinical psychology and psychiatry, similar to the politicization documented in social and political psychology (Duarte et al., 2015; Tetlock, 2020). Second, the manuals promote argumentation from authority. Specifically, they present the interpretations of those who align with the broad trauma model as objective facts, while omitting the experiences of those with different perspectives. From a professional perspective, justifying a scientific or clinical position with a sensitive personal experience exploits empathy to circumvent the procedures of evidence-based care. From a personal perspective, presenting a conceptualization based on personal experience as a scientific truth is disrespectful to the experiences of those who conceptualize their hardships differently. The term *trauma* does not belong to anyone (Haslam & McGrath, 2020). When it refers to personal experiences, its definition may be rooted in the cultural backgrounds, ideological assumptions, and personal preferences. In scientific or clinical discourse, however, determining the definition, scope, consequences, or treatments of trauma is a matter of reasoning, rather than respect, empathy, or ideology.

## The Risk of Pathologizing Normality and Inefficient use of Resources

Recommendations for prophylactic trauma assessments and overtreatment of subclinical problems can have adverse consequences. First, conducting trauma assessments when not clinically indicated may be intrusive for care recipients and violate their right not to be subjected to unnecessary care. From an ethical perspective, seeking information beyond what is necessary for providing care violates fundamental ethical principles (EFPA, 2005). From a clinical perspective, unnecessary questioning may lead to treatment fatigue (Heckman et al., 2015). Moreover, some interventions based on the trauma model may lead to iatrogenic effects, especially when applied to individuals who have not been clinically traumatized (Lilienfeld, 2007). These failures may go undetected by clinicians who overestimate the prevalence of trauma and misinterpret poor therapeutic progress as a result of past traumatization rather than the intervention itself (Lilienfeld, 2007). From a public health perspective, the proposed introduction of preventive trauma assessments and the call for widespread trauma treatment may strain societal resources allocated for mental healthcare, ultimately leading to the undertreatment of those with severe conditions.

## The Risk of Harming Healthcare users by replacing diversity with the trauma model

The manuals encourage mental health service users to adopt the broad trauma model as *the* explanation for their hardships. While a conceptualization based on social determinism may benefit some service users, it may be harmful to others. After all, there are a variety of effective therapies, and multiple explanations of distress may be beneficial (Mulder et al., 2017). Even for PTSD, therapies avoiding the trauma perspective can be effective and may reduce treatment-related distress and dropout rates (Eads & Lee, 2019; Frost et al., 2014; Imel et al., 2013). Similarly, in academia, it is desirable from the perspective of epistemic plurality to include those who find comfort in or identify with the broad trauma models as well as those who do not (Duarte et al., 2015; Kirmayer, 2012).

The arguments presented in the manuals for the trauma perspective may often be outweighed by arguments against the trauma model. Rather than promoting the interpretation of present hardships as the result of traumatization, it may be beneficial to loosen the perceived connection between patient’s presence and his or her past traumas. Framing current experiences as the result of past traumas can underexpose present factors and diminish care recipients'sense of agency, requiring them to “accept that they were powerless so that we can then empower them through their recovery” (Lester, 2013). In addition, patients’ relationships with significant others or society itself may be distorted by assuming their problems were caused by the actions of others. Indeed, traumatization is more common when an event is interpreted as interpersonal atrocity than when the same event is narrated as a naturally occurring phenomenon (Cromer & Smyth, 2010). Positive reappraisal rather than blaming society facilitates post-traumatic growth (Henson et al., 2021). Finally, the mental health consequences of an adverse event are proportional to *event centrality*: the degree to which a trauma is construed as a central part of identity and current life (Boals & Schuettler, 2011).

## The Risk of Iatrogenic Harm by Developing a Culture of Vulnerability

Presenting traumatization as the default response to adversity can lead to a culture in which adopting a victim identity becomes the default response to stress, which can significantly affect our lived experiences. The risk of traumatization is influenced by expectations and social learning. For instance, expectations may cause an event to be perceived as traumatizing due to a perceived danger (Avina & O’Donohue, 2002). Indeed, individuals who adopt the broad trauma perspective are more likely to interpret an experience as traumatizing and be negatively affected by it (Jones & McNally, 2022). Moreover, females are more likely to perceive social events as traumatizing (Frissa et al., 2016), which may reflect the impact of the construct of *gender trauma* on women’s lived realities. The effect of framing an event as a threat versus a challenge on the perception and physiological response to stressors has been experimentally confirmed (Bonanno, 2021; Tomaka et al., 1997). In general, framing human hardships as traumata may give rise to feelings of disability and helplessness. Therefore, the assumption that adverse experiences are best to be interpreted as trauma may become a self-fulfilling prophecy. Encouraging the public to interpret adverse experiences as signs of psychopathology may inflate the observed prevalence of mental disorders and exacerbate their true prevalence, according to the prevalence inflation hypothesis (Foulkes & Andrews, 2023). Similarly, promoting the trauma interpretation can increase the perceived spread of trauma and have an iatrogenic effect on individuals who would otherwise maintain their health and well-being by relying on resilience and intuitive coping strategies, had they not been convinced they are traumatized.

## Limitations

The presented study is primarily limited by its aim and scope, which only allow to comment on some aspects of the selected manuals. Specifically, we do not discuss the benefits of the trauma-informed movement that have already been thoroughly discussed in the manuals themselves and elsewhere (e.g., Berliner & Kolko, 2016; Reeves, 2015; Sweeney & Taggart, 2018; Sweeney et al., 2016). Our study is also limited by the selection of two clinical manuals, although the practices and risks identified by our study are often more pronounced in other literature promoting the broad trauma model (e.g., in the cultural rather than clinical realm, gray literature, or resources for the public). However, we see this as a strength rather than a weakness for two reasons. First, by analyzing influential manuals written with clinical expertise and scientific rigor, we avoid petty criticism of subjective opinions. Second, the selection of literature presenting the broad trauma model as universal and unchallenged corresponds to our interest in the process of asserting the dominance of the broad trauma model in the clinical field. Finally, the results of the study are inherently influenced by our own decisions, views, and biases. Indeed, CDA itself is not immune to the same discourse practices it is attempting to uncover (Billig, 2008). Such positionality, however, does not strip the analyses presented of their value in demonstrating the positionality of trauma-informed guidelines that present themselves as neutral and uncontroversial.

## Implications

Several aspects of the recommendations proposed by the manuals should be clarified before their implementation to routine clinical care. The ethical aspects of screening for trauma and encouraging patients to adopt a particular model should be elucidated. The cultural validity of interpreting specific gender, ethnic, or historical realities as traumatizing should be considered, because these interpretations may not be universally valid. The compatibility of the broad trauma model with evidence-based treatments based on other conceptualizations of psychopathology should be assessed. The efficacy and cost-effectiveness of the individual recommendations should be examined, particularly with regard to the costs of reimplementing existing procedures on one hand and requesting clinicians to adopt ideological assumptions on the other. Further research should address the interplay between the constructs of psychopathology and experiencing mental health problems. For example, it should be explored how lived experiences of human hardships may be affected by interpreting them as trauma before mental health service users are actively encouraged to conceptualize them as such.

## Footnotes

1 For instance, the DSM-5 includes the following criteria: “Exposure to actual or threatened death, serious injury, or sexual violence in one (or more) of the following ways: 1. Directly experiencing the traumatic event(s). 2. Witnessing, in person, the event(s) as it occurred to others. 3. Learning that the traumatic event(s) occurred to a close family member or close friend. In cases of actual or threatened death of a family member or friend, the event(s) must have been violent or accidental. 4. Experiencing repeated or extreme exposure to aversive details of the traumatic event(s) (e.g., first responders collecting human remains: police officers repeatedly exposed to details of child abuse)” (American Psychiatric Association, 2013, p. 271).

3 In one case, the SHITF states that “relying on seclusion and restraint (…), clinicians may learn to rely on power rather than their relational capacity to engage collaboratively, *particularly where trauma-uninformed organizations place a high priority on risk management*” (emphasis added). While trauma-uninformedness is explicitly mentioned here, there is no explanation or evidence provided why relying on seclusion and restraint should have negative impact particularly in “trauma-uninformed organizations.” Of the substantial evidence cited to document the general negative effects of seclusion and restraint, no evidence is provided to suggest that it is trauma-informedness rather than avoiding seclusion and restraint, what may protect from the adverse effects of these methods.

4 The vignettes were “inspired by an exploration of people’s experiences of iatrogenic harm” (SHIFT, p 331).

5 Given the multicausality of human suffering, virtually any event can be interpreted as having"lasting adverse effects on individual's functioning or well-being", because all adversities are causally linked to individuals'personal histories, as well as the ancient events shaping individual's society through historical power struggles, and even the prehistoric evolutionary pressures shaping individual's DNA. With no explicit criteria, inclusion in the trauma or survivor category depends solely on the observer's interpretation.
